# A Probabilistic Liquefaction Hazard Analysis: Case Studies from the Marmara Region

**DOI:** 10.1007/s10706-024-03042-6

**Published:** 2025-01-11

**Authors:** Ilya Sianko, Zuhal Ozdemir, Iman Hajirasouliha, Kypros Pilakoutas

**Affiliations:** School of Mechanical, Aerospace and Civil Engineering, The University of Sheffield, Sheffield, UK

**Keywords:** Liquefaction, Probabilistic liquefaction hazard analyses, Monte-Carlo (MC) simulations, Marmara region

## Abstract

Earthquake induced soil liquefaction poses a significant threat to buildings and infrastructure, as evidenced by numerous catastrophic seismic events. Existing approaches of regional liquefaction hazard assessment predominantly rely on deterministic analysis methods. This paper presents a novel Probabilistic Liquefaction Hazard Analysis (PLHA) framework based on Monte-Carlo (MC) simulations to mitigate future seismic risks associated with liquefaction. The proposed procedure requires only publicly available data, offering accessibility and applicability in resource-constrained settings. A key feature of the procedure is its ability to deal with uncertainties in earthquake and soil parameters using distribution functions. Liquefaction potential is assessed through parameters such as Liquefaction Potential Index ($LPI$) and Liquefaction Severity (${L}_{S}$). The procedure is implemented in MATLAB as part of a broader probabilistic risk assessment framework for developing countries. The developed procedure is applied to the high risk city of Adapazari, Türkiye; an area lacking prior PLHA studies. Results are validated against observed liquefaction data from a simulated scenario event of the 1999 Kocaeli earthquake. Probabilistic liquefaction hazard maps are generated for the study area and the entire Marmara region in terms of $LPI$ and ${L}_{S}$. A novel aspect of this work is the integration of a time-dependent Probabilistic Seismic Hazard Analysis (PSHA) model into the PLHA framework. Results are compared with those predicted using the Poisson model for the Marmara region. Findings demonstrate that the developed PLHA procedure offers a robust and flexible tool for predicting seismic liquefaction hazards, providing valuable insights for loss estimation and risk mitigation planning.

## Introduction

Earthquake induced liquefaction possess a significant threat to critical infrastructure, including buildings, roads, pipelines and buried cables as evidenced by several major seismic events (e.g. Kocaeli 1999, Chi- Chi 1999 (Sonmez et al. 2008), and Tohoku 2011 (Yamaguchi 2011)). Mitigating structural damage associated with liquefaction requires identifying liquefaction-prone areas during the site selection and planning stages by developing detailed liquefaction susceptibility maps. Nevertheless, the models currently used by the insurance industry to estimate losses due to liquefaction typically rely on simplified approaches. These models apply a correction factor to losses associated with strong ground motions based on the overall liquefaction susceptibility of the region (Bird et al. 2006). Another widely used approach for assessing liquefaction hazard involves deterministic methods, where earthquake magnitude is coupled with ground shaking intensity derived from PSHA results (e.g. Youd and Idriss 2001; Cetin et al. 2004; Rahman et al. 2015; Putti and Satyam 2018). In these methods, the likelihood of liquefaction occurrence is assessed using the factor of safety, which is defined as the ratio of the Cyclic Resistance Ratio (CRR) to the Cyclic Stress Ratio (CSR). The seismic loading on soil is represented by the CSR, while the soil resistance to liquefaction is represented by the CRR, which is derived from in-situ “index” tests reflecting observed field behaviour. Common in-situ tests include: (1) the Standard Penetration Test (SPT); (2) the Cone Penetration Test (CPT); (3) in-situ shear wave velocity (${V}_{s}$) measurements; and (4) the Becker Penetration Test (Youd and Idriss 2001). While earthquake magnitude and ground shaking intensity couples are often determined probabilistically, deterministic approaches typically use single fixed values as input for evaluating liquefaction potential (Franke et al. 2016). However, a wide range of ground shaking intensities and magnitudes may occur at a site of interest, driven by multiple seismic sources, each with varying liquefaction-triggering potential. Moreover, even earthquakes of relatively small magnitudes ($M$~5) have been found to trigger liquefaction under specific conditions (Musson 1998). As a result, deterministic approaches may underestimate the liquefaction hazard. Modern performance-based design approaches require knowledge of the probability of exceedance of liquefaction severity at the given site due to all possible ground motions for a given return period (e.g. 50 years). This highlights the need for a more advanced and probabilistic approach to estimate liquefaction hazard for enhanced safety in design, loss estimation and post-event assessment studies.

Several studies (e.g. Finn and Wightman 2007; Kramer and Mayfield 2007; Juang et al. 2008; Salloum 2008) combine PSHA with liquefaction potential assessment procedures, such as the stress-based simplified procedure by Seed and Idriss (1971). These approaches typically utilise seismic hazard curves in terms of PGA alongside the disaggregation of PSHA results. This enables the consideration of the joint probability distribution of PGA and moment magnitude ${M}_{w}$ for selected earthquake scenarios; key inputs for stress-based simplified liquefaction assessment procedures. This approach was employed by Sajan et al. (2020) to assess liquefaction hazard in the Kathmandu valley, Nepal. However, such assessments are often limited by the availability of detailed seismic hazard information, as seismic hazard estimates and deaggregation of results are available only for a few return periods and a reference soil condition. Therefore, this can restrict the use of performance-based earthquake engineering procedures for liquefaction potential evaluation (Kramer and Elgamal 2001; Kramer and Mayfield 2007). To address these challenges, Makdisi and Kramer (2024) proposed an enhanced probabilistic liquefaction hazard analysis (PLHA) methodology designed to integrate seamlessly into the PBEE framework. Their approach accounts for uncertainties in earthquake ground motions, soil resistance, and triggering mechanisms.

To address the research gaps highlighted above, this paper introduces a new fully probabilistic liquefaction hazard analysis (PLHA) procedure that can consider all possible potential ground shaking events and associated magnitudes through a stochastic Monte-Carlo (MC) simulation process. This procedure utilises a practical MC-based PSHA tool developed in a previous study (Sianko et al. 2020), which uses publicly available data on a region’s seismo-tectonic structure, seismicity and geology to generate synthetic earthquake catalogues. MC-based PHLA approaches have recently been integrated into modern seismic hazard and risk analysis software, such as OpenQuake (Pagani et al. 2014) and R-CRISIS (Ordaz et al. 2022). These have already been applied to mainland Portugal and a case study area in Mexico by Yilmaz et al. (2021) and Ordaz et al. (2023), respectively. Unlike previous works, the procedure developed in this study is implemented in MATLAB as part of a probabilistic risk assessment framework for developing countries. The proposed PLHA procedure aims to estimate the return period for specific liquefaction hazard levels, rather than simply indicating the occurrence or non-occurrence of liquefaction for a specified earthquake scenario. This study also examines various liquefaction potential prediction methods, such as Liquefaction Potential Index ($LPI$) and Liquefaction Severity (${L}_{S}$). To validate the accuracy of the developed PLHA tool, the city of Adapazari and Marmara region of Türkiye are selected as case studies for large and small-scale applications, respectively. For the Marmara region, an indicative PLHA map is prepared using freely available slope based ${V}_{s30}$ data to represent soil conditions. A more detailed PLHA study is performed for Adapazari using borehole data from multiple sources. Additionally, a parametric study is performed to investigate the effect of stress-reduction factor *r*_*d*_ on liquefaction prediction parameters and the distribution of earthquake magnitudes contributing to the liquefaction hazard. For the first time, alongside the traditional Poisson model, a time-dependent probabilistic seismic hazard analysis (PSHA) model is used to predict liquefaction hazard in the Marmara region.

## Deterministic Liquefaction Potential Assessment

Deterministic liquefaction hazard is normally assessed by comparing soil liquefaction resistance against earthquake demand. The simplified procedure originally developed by Seed and Idriss (1971) is commonly used for assessing the cyclic stress ratio (CSR), which represents earthquake demand for liquefaction potential. In this procedure, the safety factor against liquefaction (${F}_{S}$) is calculated as the ratio of the cyclic resistance ratio (CRR) to the CSR for a given layer of soil at depth z:

1 $$F_{S} = \frac{CRR}{{CSR}}$$

The condition ${F}_{S}\ge 1$ indicates non-liquefiable soil profiles, whereas ${F}_{S}<1$ indicates liquefiable soil profiles.

According to Seed and Idriss (1971), CSR can be expressed by:

2 $$CSR = 0.65\left( {\frac{{a_{max} }}{g}} \right)\left( {\frac{{\sigma_{v} }}{{\sigma_{v}^{\prime } }}} \right)r_{d}$$

where ${a}_{max}$ is the horizontal PGA (Peak Ground Acceleration) in $g$; $g$ is the acceleration due to gravity; ${\sigma }_{v}$ is the total stress at depth $z$; $\sigma_{v}^{\prime }$ is the effective stress at depth $z$; and *r*_*d*_ represents the average value of shear stress reduction factor. Iwasaki (1986) proposed that *r*_*d*_ roughly linearly decreases with depth. Due to its simplicity, this procedure is widely adopted in general practice. For standard structures, *r*_*d*_ can be calculated using relationships provided by Liao and Whitman (1986):

3 $$r_{d} \left( z \right) = \left\{ {\begin{array}{*{20}l} {1.0 - 0.00765z } \hfill & {for\;\;z \le 9.15\;{\text{m}} } \hfill \\ {1.174 - 0.0267z} \hfill & {for\;\;9.15 < z \le 23\;{\text{m}}} \hfill \\ \end{array} } \right.$$

In these equations, *r*_*d*_ is assumed to be independent of earthquake magnitude. Nonetheless, in liquefaction hazard analysis, *r*_*d*_ is expected to be influenced by earthquake magnitude, and will determine the minimum magnitude capable of triggering liquefaction. Cetin and Seed (2004) proposed the following relationship to estimate *r*_*d*_ as a non-linear function of $d$, ${M}_{w}$,${a}_{max}$ and ${V}_{s12}$ (the average shear wave velocity in top 12 m):

for *z* < 20 m

4 $$r_{d} = \frac{{\left[ {1 + \frac{{ - 23.013 - 2.949a_{max} + 0.999M_{w} + 0.0525V_{s12} }}{{16.258 + 0.201e^{{0.341\left( { - z + 0.0785V_{s12} + 7.586} \right)}} }}} \right]}}{{\left[ {1 + \frac{{ - 23.013 - 2.949a_{max} + 0.999M_{w} + 0.0525V_{s12} }}{{16.258 + 0.201e^{{0.341\left( {0.0785V_{s12} + 7.586} \right)}} }}} \right]}} \pm \sigma_{{\varepsilon_{{r_{d} }} }}$$

for *z* ≥ 20 m

5 $$r_{d} = \frac{{\left[ {1 + \frac{{ - 23.013 - 2.949a_{max} + 0.999M_{w} + 0.0525V_{s12} }}{{16.258 + 0.201e^{{0.341\left( { - 20 + 0.0785V_{s12} + 7.586} \right)}} }}} \right]}}{{\left[ {1 + \frac{{ - 23.013 - 2.949a_{max} + 0.999M_{w} + 0.0525V_{s12} }}{{16.258 + 0.201e^{{0.341\left( {0.0785V_{s12} + 7.586} \right)}} }}} \right]}} - 0.0046 \left( {z - 20} \right) \pm \sigma_{{\varepsilon_{d} }}$$

where

6 $$\sigma_{{\varepsilon_{{r_{d} }} }} = z^{0.85} 0.0198\quad for\; z < 12\;{\text{m}}$$

7 $$\sigma_{{\varepsilon_{{r_{d} }} }} = 12^{0.85} 0.0198\quad for\;z \ge 12\;{\text{m}}$$

In the above equations $\sigma_{{\varepsilon_{{r_{d} }} }}$ is the standard deviation of${r}_{d}$.

$CRR$ in Eq. (1) is usually calculated using soil parameters obtained from cone penetration tests (CPT) or standard penetration tests (SPT). However, Andrus and Stokoe (2000) proposed a different approach for calculating $CRR$ using the shear-wave velocity:

8 $$CRR=\left[0.022{\left(\frac{{V}_{s1,\text{cs}}}{100}\right)}^{2}+2.8\left(\frac{1}{{V}_{s1}^{*}-{V}_{s1,\text{cs}}}-\frac{1}{{V}_{s1}^{*}}\right)\right]\times MSF$$

9 $$V_{{s1,{\text{cs}}}} = V_{s1} K_{FC} = V_{s} \left( {\frac{{P_{a} }}{{\sigma_{v}^{\prime } }}} \right)^{0.25} \times K_{FC}$$

where ${V}_{s1,\text{cs}}$ stands for the stress-corrected shear wave velocity; ${V}_{s1}$ represents the overburden-stress-corrected shear-wave velocity; ${P}_{a}$ shows a reference stress (typically 100 kPa); ${V}_{S1}^{*}$ defines an upper limit for cyclic liquefaction occurrence varying between 200 and 215 m/s depending on the fines content (FC in %) of the soil; and ${K}_{FC}$ which shows an adjustment factor for FC is defined as follows (Juang et al. 2001):

10 $$K_{FC} = \left\{ {\begin{array}{*{20}l} {1.0 } \hfill & {for\;FC \le 5\% } \hfill \\ {1.0 + \left( {{\text{FC}} - 5} \right)T} \hfill & {for\;5\% < FC < 35\% } \hfill \\ {1 + 30T} \hfill & {for\;FC \ge 35\% } \hfill \\ \end{array} } \right.$$

where

11 $$T=0.009-0.0109\left(\frac{{V}_{s1}}{100}\right)+0.0038{\left(\frac{{V}_{s1}}{100}\right)}^{2}$$

In Eq. (8), $MSF$ represents the magnitude scaling factor, which can be calculated as follows (Youd and Idriss 1997):

12 $$MSF={\left(\frac{{M}_{w}}{7.5}\right)}^{-2.56}$$

$MSF$ reflects the number of significant cycles, and therefore, can be assumed to be related to the ground motion duration.

According to Maurer et al. (2014), severe liquefaction will generally occur if the liquefiable layer is thick and located close to the surface, and ${F}_{S}$ calculated for this layer is far less than 1.0. Juang et al. (2005) found that Eq. (8) is conservative, over-predicting liquefaction occurrence, and proposed a multiplication factor of 1.4 for $CRR$.

Data needed for ${V}_{s}$ in the $CRR$ calculations (Eqs. 8 and 9) are not commonly available from public domain ground investigations and may not necessarily be available across the entire study area. Thus, in many cases, geo-statistical techniques are required for its determination. Two approaches are proposed to approximate ${V}_{s}$ from more readily available data. The first approach is based on the ${V}_{s30}$ value, which is obtained by averaging ${V}_{s}$ values for the top 30 m of soil to determine the shear wave velocity for all soil layers. The main drawback of this approach is that liquefaction potential is highly influenced by the top layers of the soil, which usually have lower ${V}_{s}$ values than ${V}_{s30}$ thus this leads to under-prediction of liquefaction hazard in calculations. Assuming a constant ${V}_{s30}$ value for all soil layers might cause an overestimation of ${V}_{s}$, $CRR$ and ${F}_{S}$ at the top layers, which can result in an underestimation of liquefaction hazard. On the other hand, worldwide ${V}_{s30}$ estimates are available through open-access web-based US Geological Survey Global ${V}_{s30}$ Map Server (USGS). This makes the ${V}_{s30}$ approach suitable for studies in regions where soil data is restricted or not available.

As a second approach, empirical equations proposed by Boore et al. (2011) can be used to estimate ${V}_{S10}$ and ${V}_{S20}$, for the average shear wave velocity across the top 10 m and 20 m of soil, respectively, to calculate $CRR$ using Eq. (8):

13 $${V}_{s10}={10}^{\left(\frac{\text{log}{V}_{s30}-0.042062}{1.0292}\right)}$$

14 $${V}_{s20}={10}^{\left(\frac{\text{log}{V}_{s30}-0.025439}{1.0095}\right)}$$

From these equations, the average shear wave velocity between top 10 m and 20 m of soil (${V}_{s(10-20)}$) can be obtained as follows:

15 $${V}_{s(10-20)}=\frac{1}{\frac{2}{{V}_{s20}}-\frac{1}{{V}_{s10}}}$$

Although ${F}_{S}$ of a soil layer can be estimated by means of various geotechnical parameters, it is not sufficient to assess liquefaction potential and thus it is not a practical parameter for use in liquefaction severity maps. ${F}_{S}$ can predict if a layer will liquefy or not, but it cannot predict the severity degree. To overcome these limitations, the liquefaction potential index ($LPI$) was proposed by Iwasaki et al. (1984):

16 $$LPI={\int }_{0}^{20}F w(z) dz$$

where for a single soil layer $w\left(z\right)=\left(10-0.5z\right)$, and if ${F}_{S}$ < = 1 $F=1-{F}_{S}$ and otherwise $F$=0.

In this method, the soil profile is sub-divided into a number of layers and the liquefaction potential at the surface-layer is predicted by integrating a function of the factor of safety for each soil layer within the top 20 m of soil. According to severity categories proposed by Iwasaki et al. (1982), liquefaction potential is “very low” for $LPI$ = 0; “low” for 0 < $LPI$  ≤ 5; “high” for 5 < $LPI$  ≤ 15; and “very high” for $LPI$ > 15. Several studies (e.g. Toprak and Holzer 2003; Kongar et al. 2017) have used the $LPI$ procedure to investigate appropriate thresholds for liquefaction occurrence. Based on field observations, they proposed $LPI=4-5$ as a threshold value for moderate liquefaction hazard (e.g., sand boils), whereas $LPI=12-15$ was proposed as a threshold value for major liquefaction hazard (e.g., lateral spreads).

The $LPI$ model also requires water table depth and unit weights of soil layers. If such data are not available, engineering judgment needs to be used to estimate these parameters based on information from available sources.

The liquefaction potential can also be assessed in terms of probability of liquefaction, ${P}_{L}$. Juang and Jiang (2000) extended earlier studies on the Bayesian mapping function, and found that mapping functions could be developed using the distributions of calculated ${F}_{S}$. In a different study, Juang et al. (2002) used $225$ ${V}_{s}$-based case studies from Andrus and Stokoe (2000) to obtain a Bayesian mapping function, that relates ${F}_{S}$ determined from the ${V}_{s}$-method with ${P}_{L}.$ The developed function estimates a 26% probability (${P}_{L}=0.26$) of liquefaction occurrence for equilibrium conditions, when ${F}_{S}=1$. The probability of liquefaction can be calculated by using Eq. (8) as proposed by Juang et al. (2002):

17 $${P}_{L}=\frac{1}{1+{\left(\frac{{F}_{S}}{0.73}\right)}^{3.4}}$$

In the ${P}_{L}$ method developed by Juang et al. (2002), for a given layer of soil liquefaction can still occur with some probability even for ${F}_{S}>1$, whereas in the procedure proposed by Iwasaki et al. (1984) it is assumed that no liquefaction can occur if ${F}_{S}>1$. According to Juang et al. (2002), the choice of a particular $MSF$ formula and *r*_*d*_ formulation is not critical to the Bayesian mapping function.

Sonmez and Gokceoglu (2005) replaced $F$ in Eq. (16) with ${P}_{L}$ to calculate liquefaction severity index (${L}_{S}$):

18 $${L}_{S}={\int }_{0}^{20}{P}_{L} w(z)dz$$

Table 1 shows severity classification proposed by Sonmez and Gokceoglu (2005), which also includes categories of ‘non-susceptible’ and ‘moderate’. These categories were not included in the original classification proposed by Iwasaki et al. (1984).

**Table 1:** Liquefaction severity index ${L}_{S}$ classification (Sonmez and Gokceoglu 2005)

${L}_{S}$	Description
85 ≤ ${L}_{S}$ < 100	Very high
65 ≤ ${L}_{S}$ < 85	High
35 ≤ ${L}_{S}$ < 65	Moderate
15 ≤ ${L}_{S}$ < 35	Low
0 < ${L}_{S}$ < 15	Very low
${L}_{S}$ = 0	Non-liquefied

The simplified liquefaction assessment methods were developed using post-earthquake field observations supported by in-situ tests. The deterministic procedures can be demonstrated to produce fairly accurate estimates of the liquefaction potential under a given pair of seismic parameters $$({a}_{max}$$,$${M}_{w})$$ and $${V}_{s30}$$, as shown by Kongar et al. (2017) for Christchurch in New Zealand.

## Probabilistic Liquefaction Hazard Analysis

Deterministic methods are applicable to a specific performance level or earthquake scenario and do not estimate liquefaction potential by taking into account all possible earthquake events. To overcome this limitation, probabilistic methods can be adopted to estimate liquefaction hazard. Atkinson et al. (1984) developed a PLHA procedure based on the conventional PSHA proposed by Cornell (1968). The method combines Seed and Idriss (1971) simplified method for assessing liquefaction potential with conventional PSHA method for assessing seismic hazard by modifying the latter to consider the joint probability of magnitude and acceleration. The drawback of this method is that treating uncertainties in conventional PSHA is not a trivial problem and often requires a logic tree, where the choice of weights for branches tends to be subjective. Another approach is to use readily available PSHA results such as hazard curves and deaggregation of hazard as proposed by Kramer and Mayfield (2007) and Juang et al. (2008). The problem with this approach is that hazard maps may not be available or are available only for specific return periods. To address this issue, Goda et al. (2011) proposed the use of an event-based PSHA to perform PLHA. However, in their work four Canadian cities were represented in PLHA calculations with a single location for each city, which is unrealistic for a hazard map. One of the outcomes of their study was that earthquake magnitudes as low as $M=4.5$ have non-negligible effect on the liquefaction hazard curves, and therefore, should be considered in the PLHA. In a more recent study, Green and Bommer (2019) suggested that ${M}_{min}$ to be considered in PLHA is $M=5$, agreeing with the lower limit proposed by Atkinson et al. (1984). Moreover, they suggested that the disparity in ${M}_{min}$ values can be attributed to the stress reduction (*r*_*d*_) relationship used in the analysis, as the relationship used by Goda et al. (2011) is independent of earthquake magnitude. The stress reduction (*r*_*d*_) relationship proposed by Cetin and Seed (2004) will be incorporated in the PLHA study conducted in this work. Recently, Mongold and Baker (2024) conducted a regional liquefaction hazard and risk assessment for Alameda, California, using Monte Carlo simulations by accounting for uncertainties in ground shaking, groundwater levels, soil parameters and empirical liquefaction potential index equations. The study serves as an example of performing regional liquefaction analysis with limited borehole data and highlights the importance of adopting regional probabilistic analysis.

### Methodology

Stochastic approaches such as MC simulations can be an effective way of directly incorporating PLHA into the PSHA procedure. In this method synthetic earthquake catalogues are generated by randomizing key parameters in a controlled manner to represent the future seismic behaviour of a region. In both conventional and MC-based PSHA studies, seismicity of an area can be represented by (i) a simple area source zone model and (ii) combination of the area source zone model with a fault source zone model. In the former, seismic events without identified faults are assigned to areal background source zones (BSZs) assuming that they occur randomly and distributed uniformly within BSZs. In the latter, seismicity caused by known active faults with a characteristic magnitude mainly occurs in fault source zones (FSZs). The latter model is adopted in this paper, as the seismicity and tectonic settings of the case study area (the Marmara region) can be better represented by this model.

After generating synthetic catalogues for BSZs and FSZs, the probability of exceedance (*PoE*) of liquefaction for various prediction parameters such as $LPI$ or ${L}_{S}$ can be determined at a site of interest. This can be done by using the distance between the site and a given earthquake event to determine the expected PGA at the site. For each year of synthetic catalogue, all PGA values generated by all source zones at a site are used to determine an annual maximum outcome for the liquefaction prediction parameter at that site in that year. This step is repeated for all simulations to find annual maximum outcomes and the results are combined into a single list. Annual maximum outcomes are sorted in descending order. The ${N}^{th}$ value in the sorted list gives the probability of exceedance of certain liquefaction prediction parameter. For the desired return period, $N$ can be computed as follows:

19 $$N = \left( {\frac{1}{Return\; period} \times { }Catalogue\, length \times Number \,of\, simulations} \right) + 1$$

**Fig. 1:**
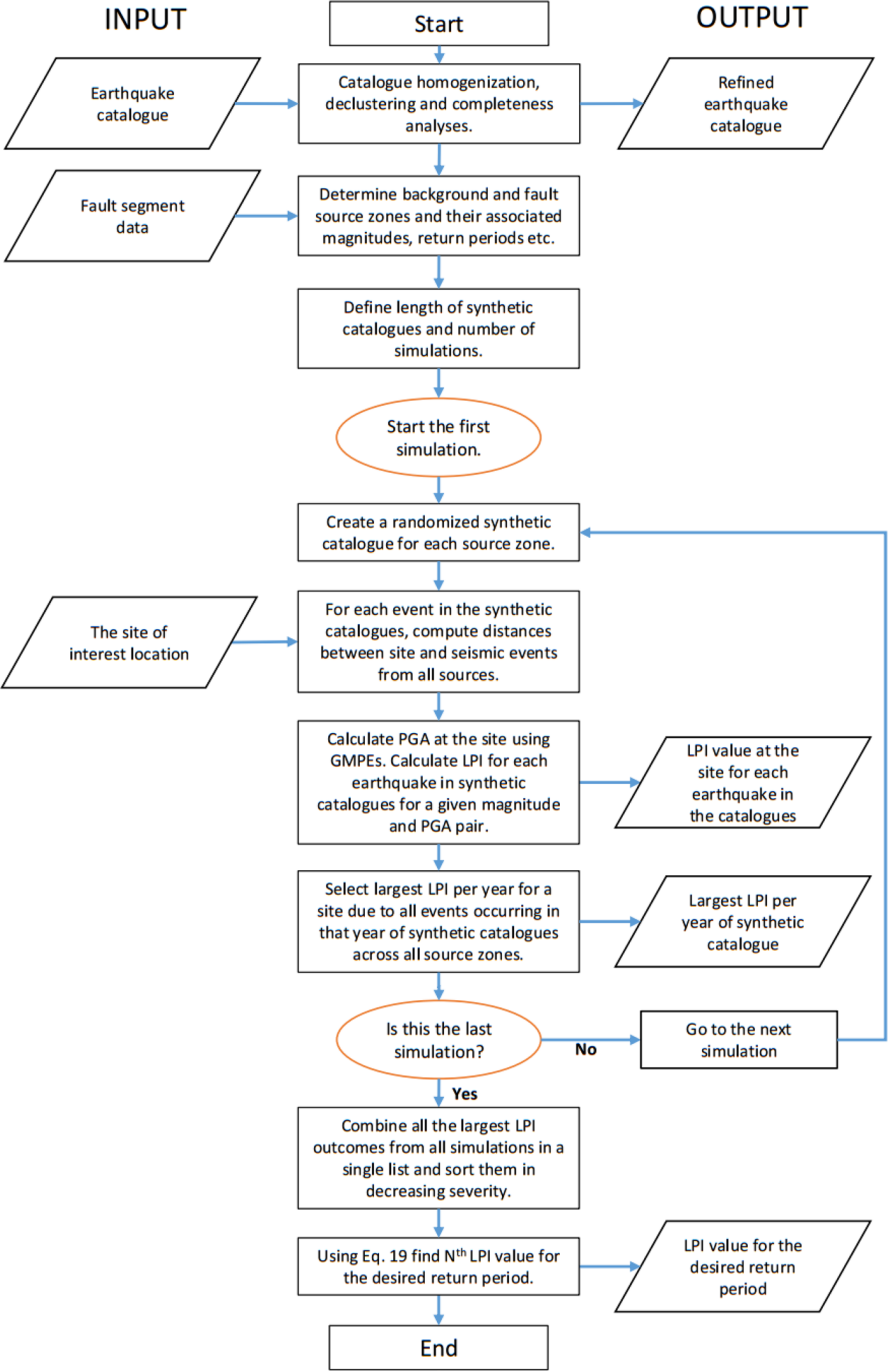
The proposed MC-based PLHA procedure using $LPI$ as liquefaction potential prediction parameter

Figure 1 shows the detailed procedure for the PLHA based on MC simulations proposed here. In this flowchart, $LPI$ is used as a liquefaction prediction parameter, but any prediction parameter can be used in the procedure.

The previous sections of the paper describe the methodology of the proposed event-based PLHA procedure. The next sections are used to validate accuracy and effectiveness of the proposed procedure by applying it to a large and small scale case studies in Türkiye.

## Large Scale Case Study: The city of Adapazari

**Fig. 2:**
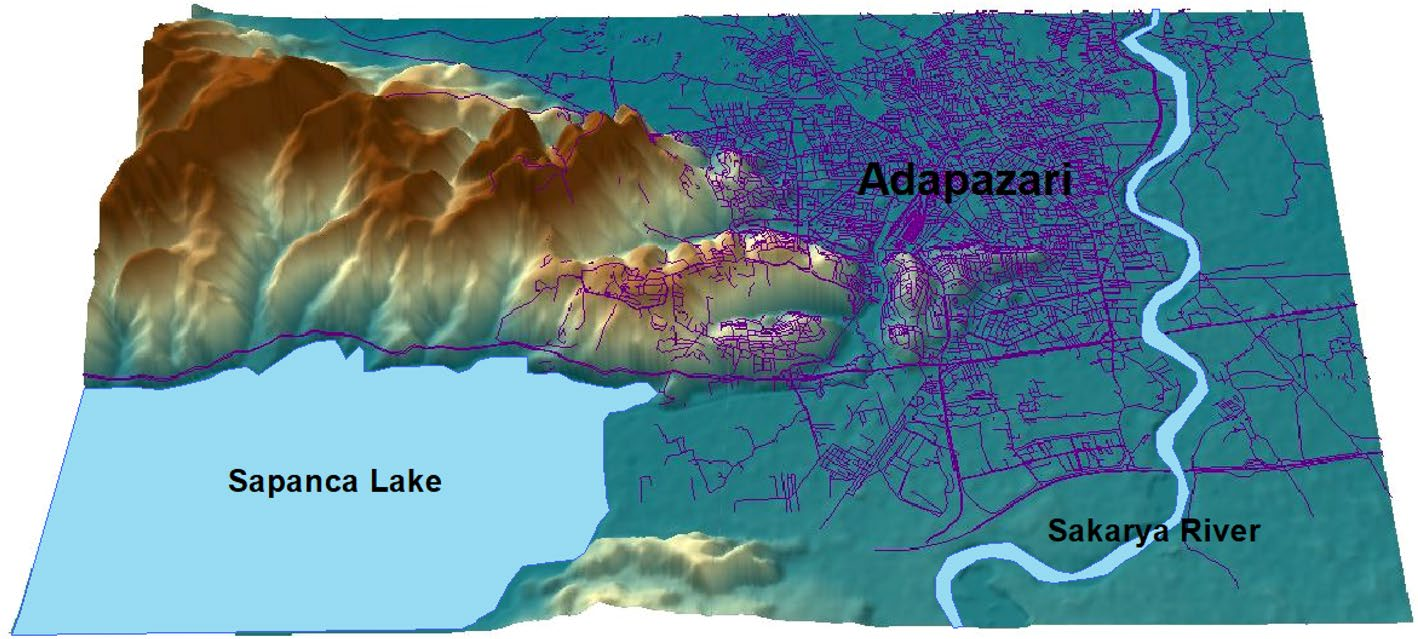
3D topographic map of the city of Adapazari and its surrounding area

**Fig. 3:**
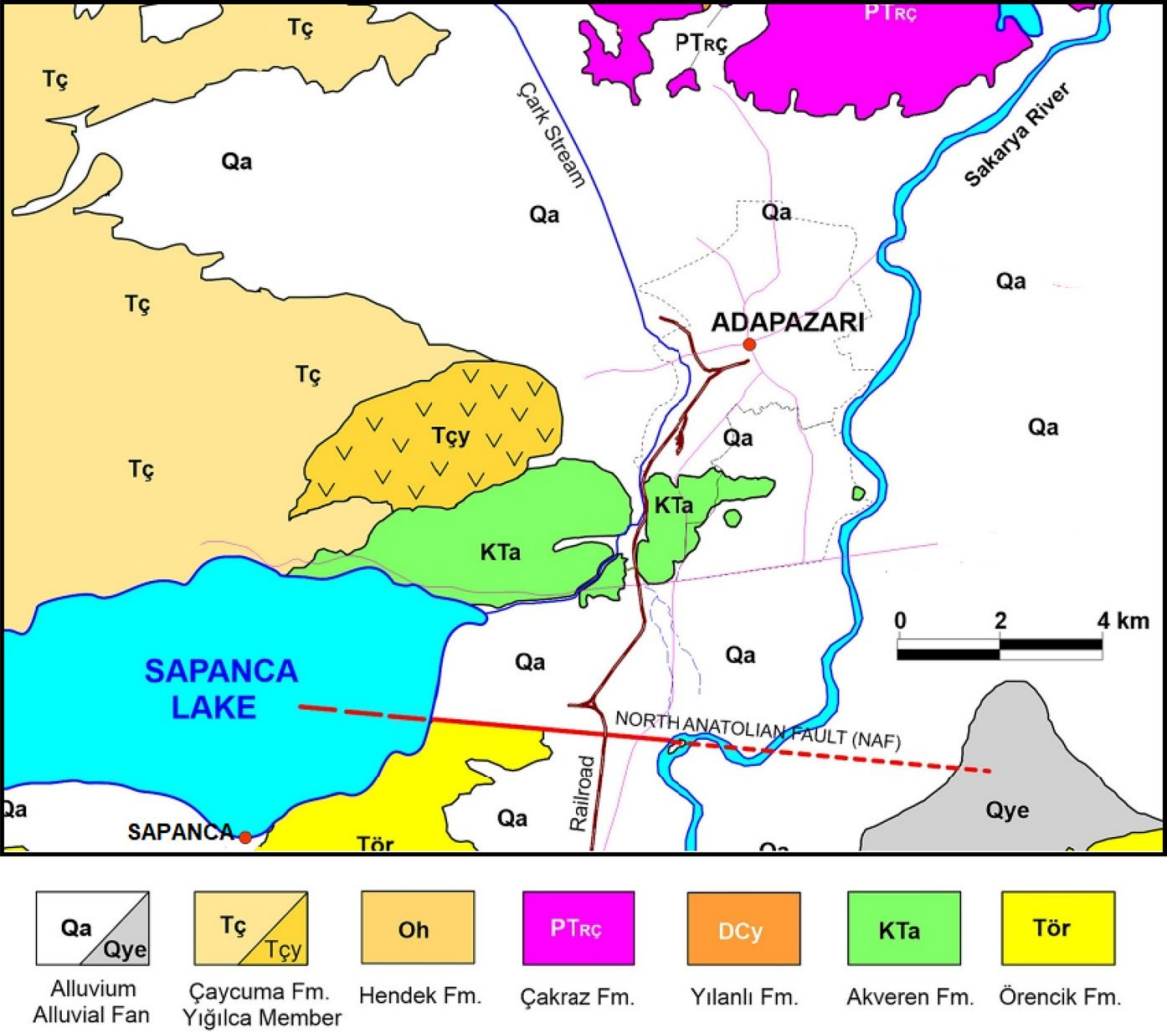
Geological map of Adapazari (Onalp et al. 2023)

The Marmara region, in north-west of Türkiye spanning Europe and Asia, lies in one of the most seismically active zones in the world. The 1999 Kocaeli earthquake with ${M}_{w}$=7.4 hit the region and resulted in significant loss of life (around 20,000 lives) and extensive damage to buildings and infrastructure. This event caused liquefaction in inland alluvial locations, along the coast of Izmit Bay and the southern coast of Sapanca Lake. In the city of Adapazari, liquefaction was characterised extensive settlement, sand boiling and lateral spreading. Due to its proximity to the Sakarya river (shown in Fig. 2), Adapazari is located on recent alluvial deposits (see Fig. 3), consisting of sand and/or silty sand, with the thickness ranging from several tens of meters to more than 300 m and potential to liquefy (Bol et al. 2010; Onalp et al. 2023). The water table level across the city is very shallow (around 1 m). Bray and Stewart (2000) provided a detailed investigation of ground failures and building damage observed in the city of Adapazari. Bray et al. (2004) conducted a detailed investigation into liquefaction using cone penetration tests (CPT) and boreholes with standard penetration tests (SPT). Their findings indicated that low-plasticity silts were the primary contributors to severe building damage during the 1999 Kocaeli earthquake. Bol et al. (2008) investigated the liquefaction potential of silty soils in Adapazari, which contributed significantly to structural damage during the 1999 Kocaeli earthquake. The researchers developed a vulnerability map based on geotechnical investigations, emphasizing the role of silt content and groundwater conditions in amplifying liquefaction risk.

Adapazari and the Marmara region are highly likely to be hit by an earthquake of ${M}_{w}$>7.3 with a 35–47% probability in the next 30 years (Murru et al. 2016). While Adapazari is a well-studied area in terms of liquefaction hazard, there is no probabilistic liquefaction hazard map for the city, hence it is selected as the first case study for the proposed procedure.

### Input Data

**Fig. 4:**
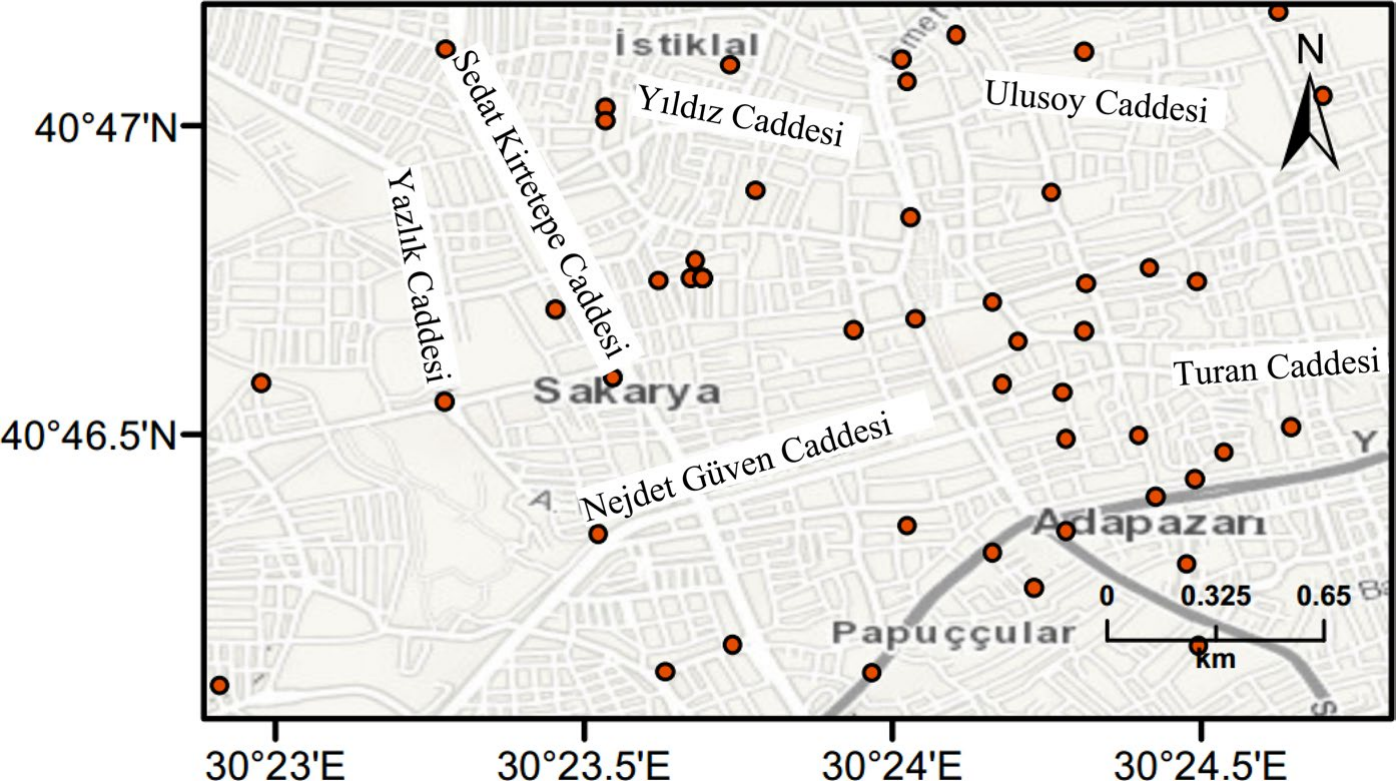
Bore logs locations across Adapazari city used in the analysis

A total of 75 borehole logs located in Adapazari were collected from different sources including PEER database (Bray et al. 2001) and Adapazari municipality. The borehole logs situated in the study area are shown in Fig. 4. Borehole data were used to determine shear wave velocity, the depth of ground water level (GWL), as well as density and fine content of soil layers across the city centre of Adapazari. Bray et al. (2004) characterized a representative soil column including a shear wave velocity profile for downtown Adapazari using data obtained from a deep borehole drilled at a specific site. The available logs vary in depth with most being up to 10 m. In the presented methodology, three depth ranges are assumed for liquefaction calculations. These are 0–5 m, 5–10 m and 10–20 m. Borehole data up to 10 m are used directly in the analysis, while data required for the 10–20 m’ range are estimated based on the data up to 10 m.

While ${V}_{s5}$ and ${V}_{s(5-10)}$ are available for some logs. For the rest the conversion equation proposed by Akin et al. (2011) is adopted to convert average SPT values to shear wave velocities as follows:

20 $$\ln V_{s} = \ln 56.1 + 0.4405\ln N + \varepsilon \sigma_{{\ln V_{s} }} ,\;{\text{where}}\;\sigma_{{\ln V_{s} }} = 0.3231$$

Once ${V}_{s10}$ is known, ${V}_{s30}$ is calculated by rearranging Eq. (13). Then, Eq. (14) is used to find ${V}_{s20}$ and finally, ${V}_{s(10-20)}$ is calculated using Eq. (15). Ground water level (GWL) from borehole logs is randomized by adding a variable value between − 0.5 m and 0.5 m sampled from uniform distribution. ${V}_{s}$ values are randomized using normal distribution with standard deviation provided in the SPT conversion equation. Stress reduction factor *r*_*d*_ is randomized with ${\sigma }_{{\varepsilon }_{{r}_{d}}}$ calculated from Eqs. (4–5) proposed by Cetin and Seed (2004).

**Fig. 5:**
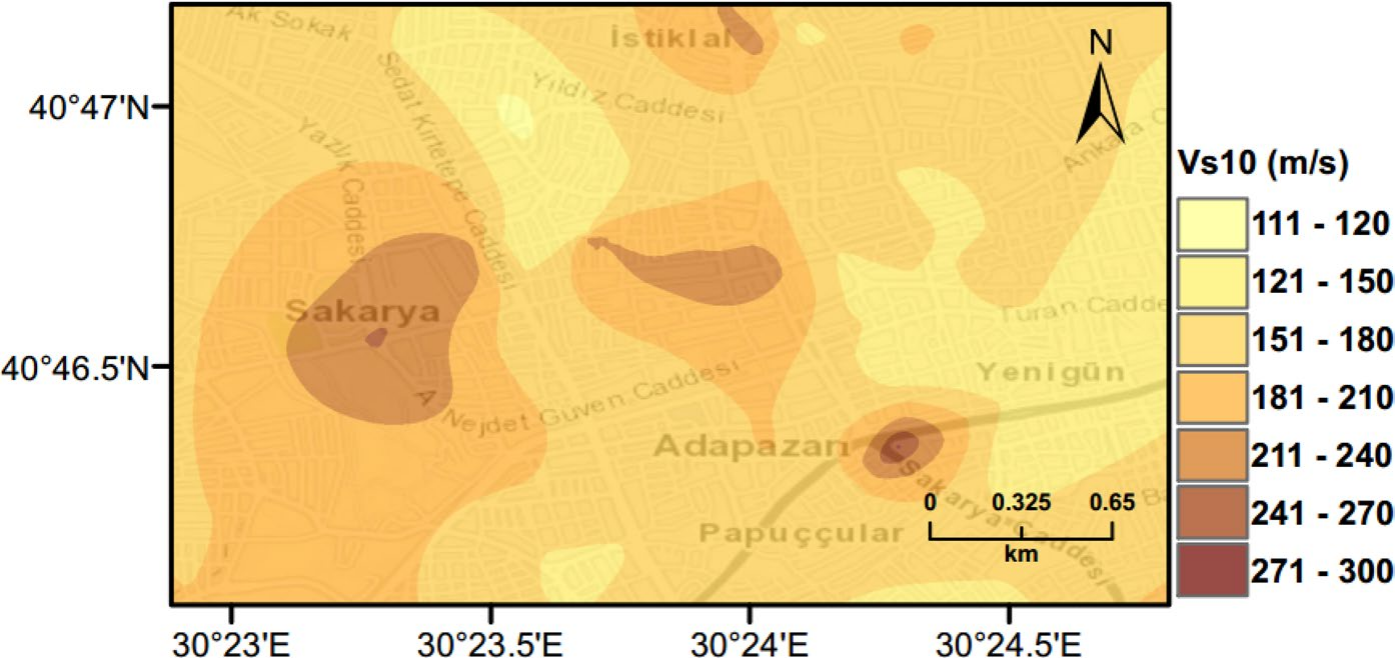
${V}_{s10}$ map for the city centre of Adapazari used in the PLHA

The uncertainties of input parameters for the liquefaction hazard calculations used in the MC method, are represented by mean values and standard deviations, are shown in Table 2. These enable synthetic catalogue generation. By using borehole logs in known locations, it is possible to estimate missing data required for the analysis of the other locations by using interpolation. Figure 5 shows an example of the obtained map for mean ${V}_{s10}$.

**Table 2:** Parameters randomized in MC simulations for liquefaction hazard calculations (metric units)

Parameter	Type of distribution	Randomization value
${V}_{s}$	Log-normal distribution	${\sigma }_{\text{ln}{V}_{s}}=0.3231$
GWL	Uniform distribution	${\sigma }_{GWL}=\pm 0.5$
${r}_{d}$	Normal distribution	${\sigma }_{{\varepsilon }_{{r}_{d}}}$
Soil density	Normal distribution	${\sigma }_{soil}=$ 0.3
$\text{FC}$	Normal distribution	${\sigma }_{fc}=$ 18.9

The MC based PSHA tool developed by Sianko et al. (2020) is utilised for the PLHA of the case study area due to its practicality and availability of Poisson and time-dependent models. The seismicity of the Marmara region is modelled by considering 25 faults source zones (FSZ) to represent large events occurring on the faults with the assigned characteristic magnitude. There are also 17 background source zones (BSZ) to represent small events occurring in the region. For more details on the adopted MC-based PSHA see Sianko et al. (2020).

### PLHA for Adapazari

**Fig. 6:**
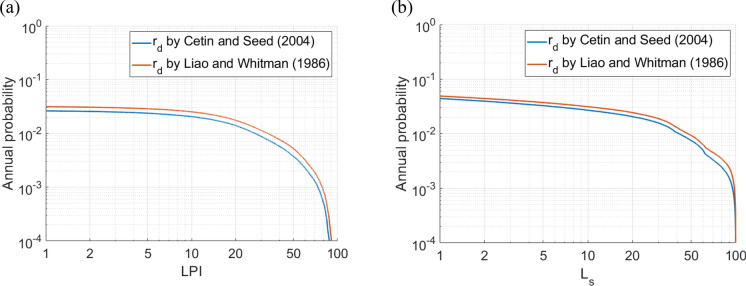
The liquefaction hazard curves for the city centre of Adapazari obtained using **a** $LPI$ and **b** ${L}_{S}$ procedures employing ${r}_{d}$ methods proposed by Liao and Whitman (1986) and Cetin and Seed (2004) for mean ${V}_{s}$ profile

**Fig. 7:**
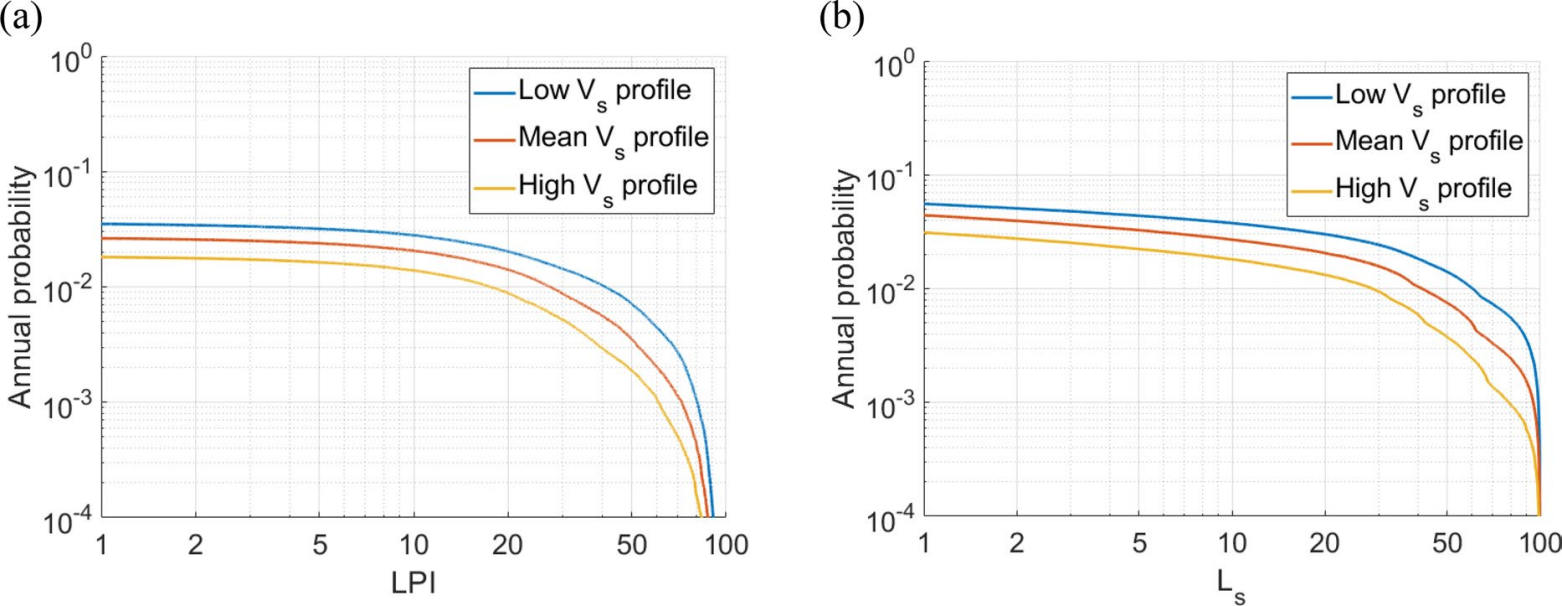
The liquefaction hazard curves for the city centre of Adapazari obtained using **a** $LPI$ and **b** ${L}_{S}$ procedures employing ${r}_{d}$ method proposed by Cetin and Seed (2004) for representative low, mean and high ${V}_{s}$ profiles

In this study, the $LPI$ method based on Eq. (16) and the ${L}_{S}$ method based on Eq. (18) are incorporated in MC-based PLHA procedure to quantify the liquefaction hazard for the city of Adapazari. *r*_*d*_ methods proposed by Liao and Whitman (1986) and Cetin and Seed (2004) are also integrated into the procedure. Figure 6 shows seismic hazard curves for the mean ${V}_{s}$ profile developed for the city centre of Adapazari using (a) $LPI$ and (b) ${L}_{S}$ procedures. It can be observed that the ${r}_{d}$ method proposed by Liao and Whitman (1986) provides more conservative predictions than that proposed by Cetin and Seed (2004), leading to a higher liquefaction hazard for the corresponding return period. In Fig. 7 seismic hazard curves are presented for low, mean and high ${V}_{s}$ profiles for the city centre of Adapazari. The mean ${V}_{s}$ profile plus or minus one standard deviation gives high or low profiles, respectively. The results clearly indicate the significant influence of ${V}_{s}$ profiles on liquefaction hazard.

**Fig. 8:**
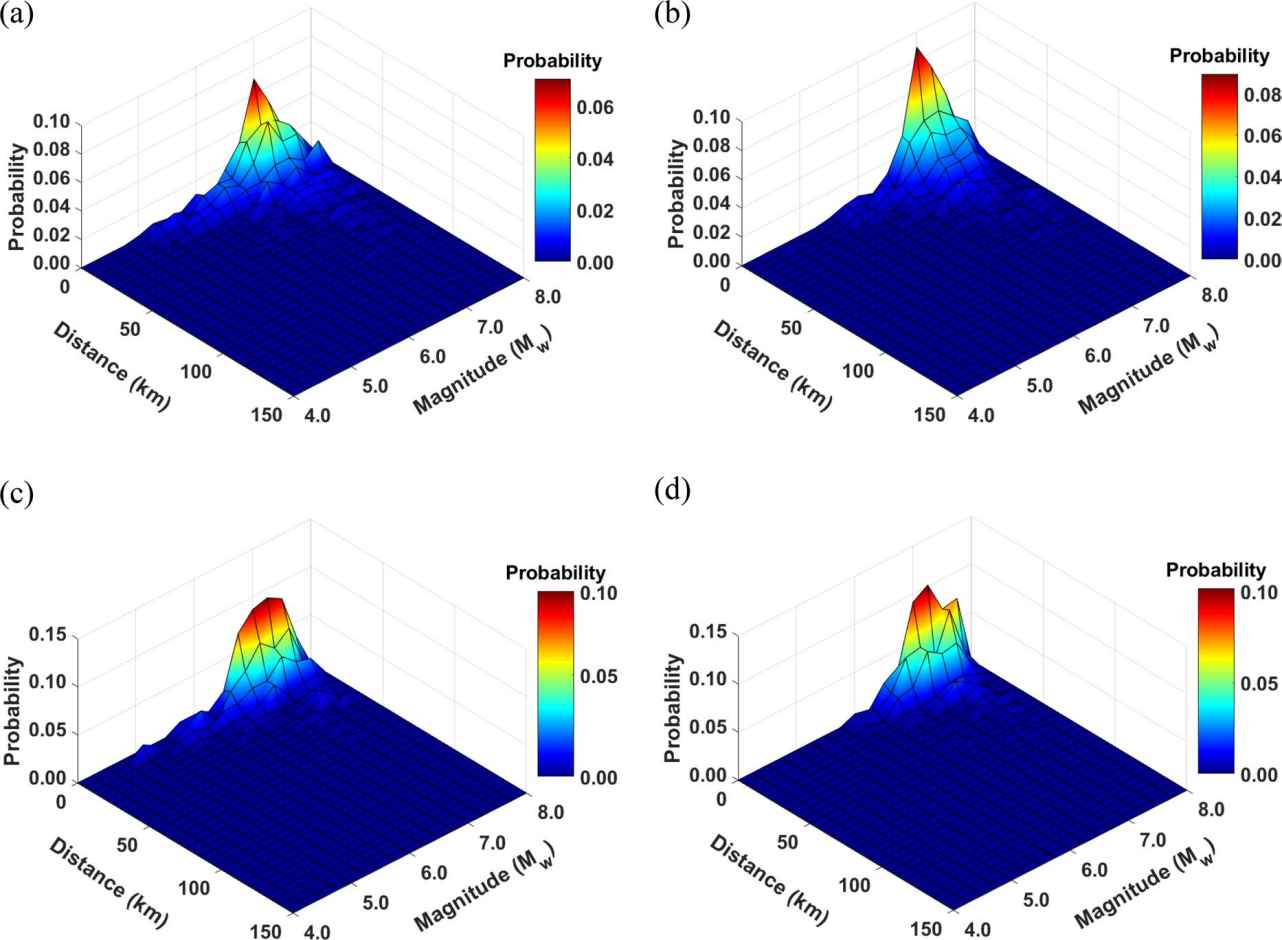
Disaggregation of liquefaction hazard for target $LPI$ for two different return periods and stress reduction factor calculation methods using mean profile: **a** APoE = 1/475, $LPI$ = 67.8, ${r}_{d}$ method: Liao and Whitman (1986), **b** APoE = 1/475, $LPI$ = 61.2, ${r}_{d}$ method: Cetin and Seed (2004), **c** APoE = 1/2475, $LPI$=85.2, ${r}_{d}$ method: Liao and Whitman (1986), and **d** APoE = 1/2475, $LPI$=81.8, ${r}_{d}$ method: Cetin and Seed (2004)

Deaggregation of results is carried out to identify earthquake magnitude and distance values that contribute to the liquefaction hazard in terms of $LPI$ at the case study area for a given return period. As opposed to the conventional PSHA method (Cornell 1968), the proposed MC-based PLHA procedure can be directly used to identify design earthquakes at sites of interest. It can be done by extracting all earthquakes from the generated synthetic catalogues’ events that produce an $LPI$ value (with some tolerance level) calculated for the desired annual probability of exceedance (APoE), as shown in the flowchart (Fig. 1). Then, a 3D surface map with the third dimension showing the probability of the events is obtained for various magnitude-distance pairs. Design earthquakes can be identified by finding magnitude-distance pair with highest probability (peaks in the plot). Figure 8 shows the deaggregation plots for the return periods of 475 and 2475 years for two different *r*_*d*_ methods using mean soil profiles for Adapazari. It can be seen that *M*_*w*_ ~ 7 is the most dominant magnitude for both return periods. This is due to the fact that Adapazari is in close proximity to active fault segments with a similar characteristic magnitude. It can also be noticed that the chosen *r*_*d*_ method has an effect on magnitude-distance distribution and $LPI$ value. For the same return period, $LPI$ values are smaller when *r*_*d*_ is calculated based on the method proposed by Cetin and Seed (2004). Also, this *r*_*d*_ method leads to less contribution of earthquakes with magnitude *M*_*w*_ < 6 in liquefaction hazard than that obtained by the Liao and Whitman (1986) method. These results support Green and Bommer (2019) findings that earthquakes of magnitude smaller than *M* = 5 should not be considered in liquefaction hazard calculations, contrary to Musson (1998) who recommended that lower magnitudes should not be excluded from the analysis.

**Fig. 9:**
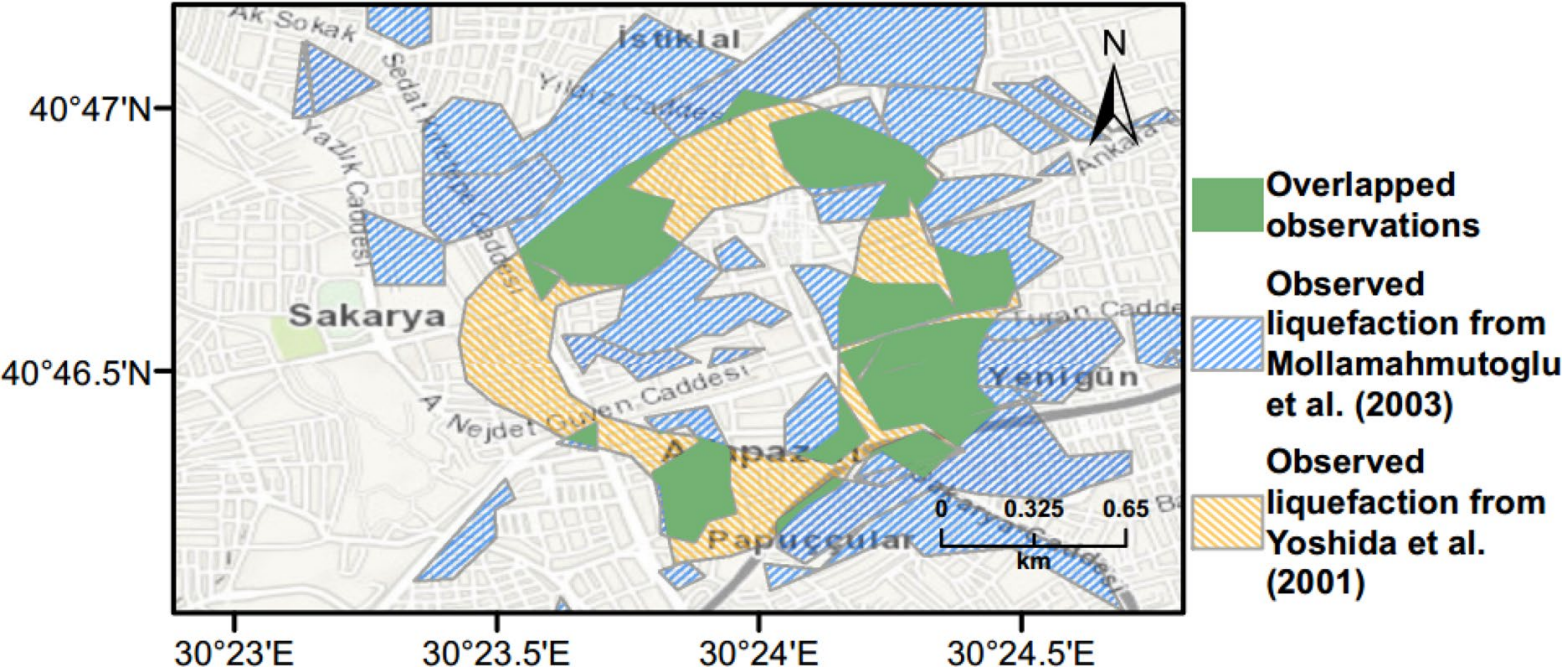
Observed liquefaction damage in Adapazari during the 1999 Kocaeli earthquake mapped from Yoshida et al. (2001) and Mollamahmutoglu et al. (2003)

Previous field studies by Yoshida et al. (2001) and Mollamahmutoglu et al. (2003) identified liquefied areas following the 1999 Kocaeli earthquake as shown in Fig. 9. As one can observe from this figure, the agreement between the two studies in terms of observed liquefaction is relatively poor. This may be attributed to the collapse of a large proportion of buildings in the city during the earthquake, which made it difficult to determine the occurrence of liquefaction.

**Fig. 10:**
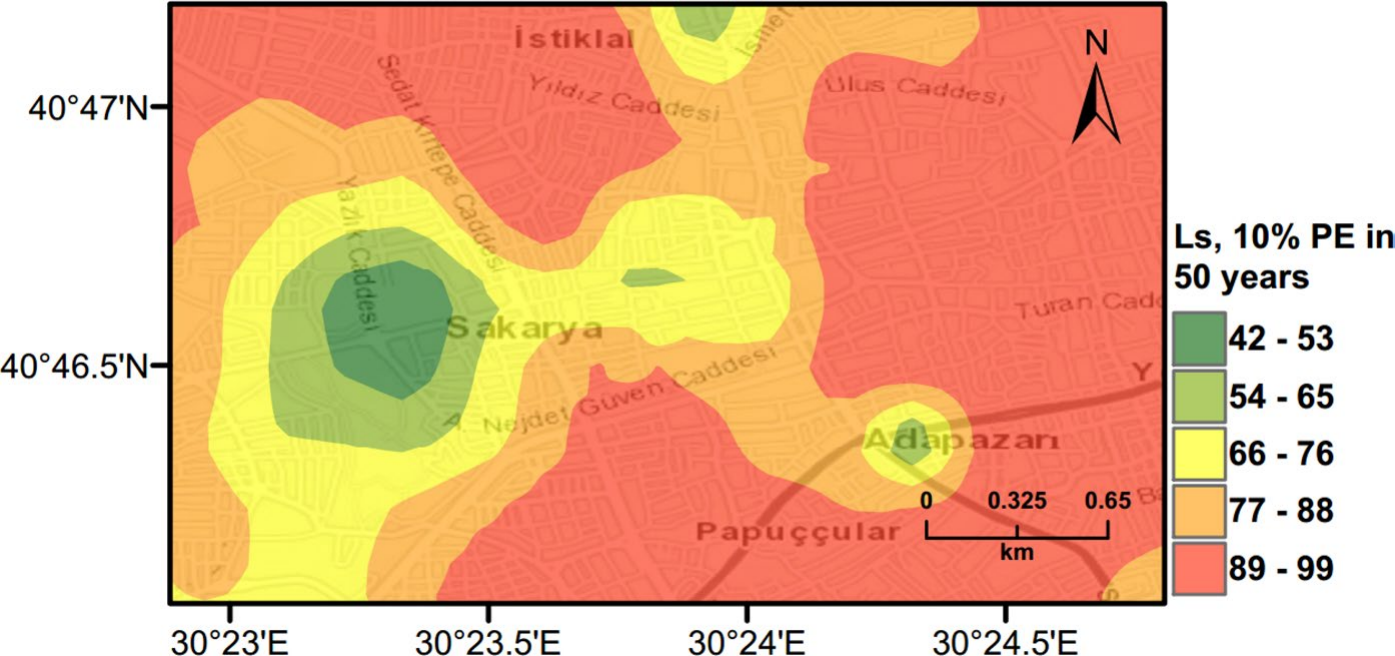
Probabilistic liquefaction hazard map in terms of ${L}_{S}$ for the central part of Adapazari city with a return period of 475 years

**Fig. 11:**
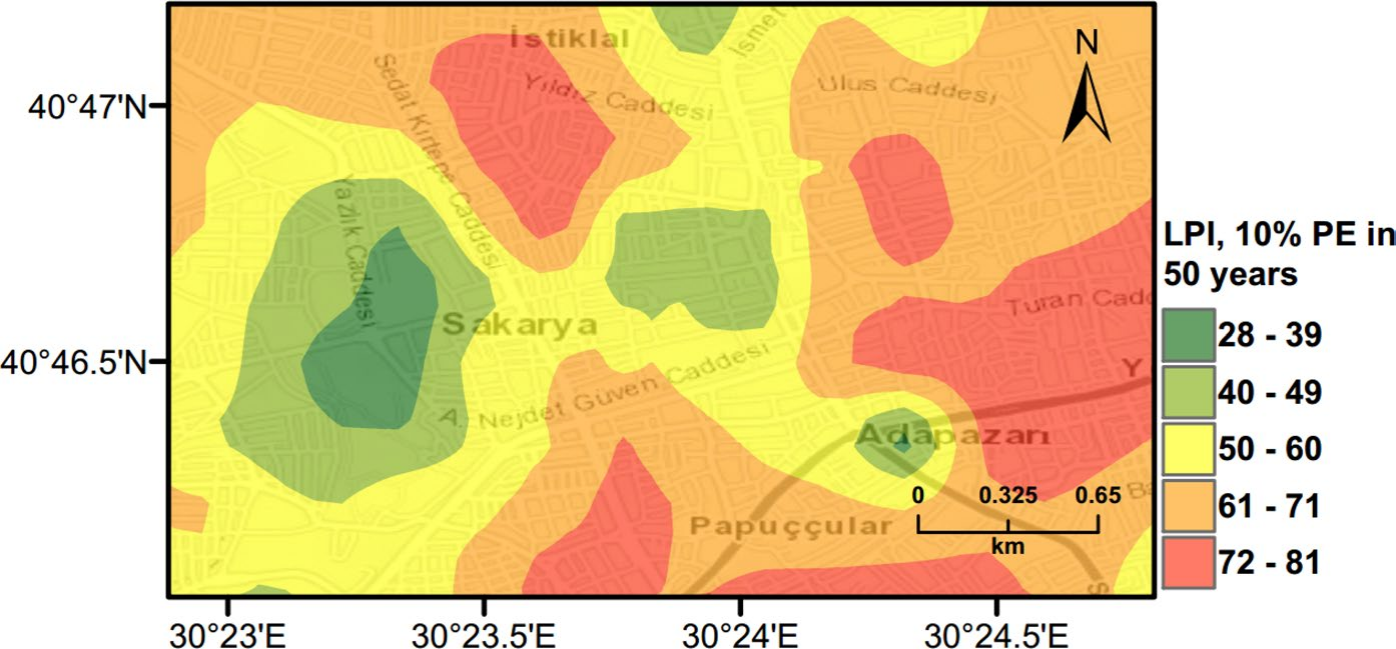
Probabilistic liquefaction hazard map in terms of $LPI$ for the central part of Adapazari city with a return period of 475 years

**Fig. 12:**
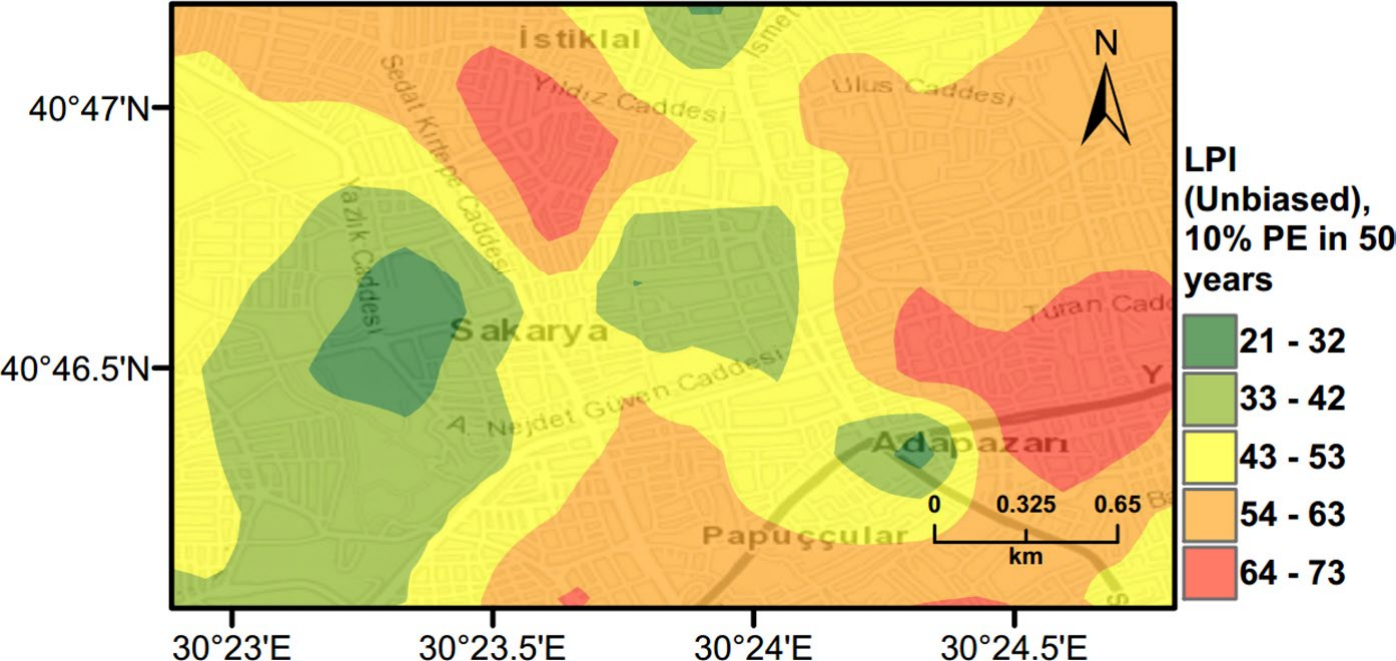
Probabilistic liquefaction hazard map in terms of unbiased $LPI$ for the central part of Adapazari city with a return period of 475 years

PLHA maps were prepared for Adapazari using the MC based PLHA procedure developed in this work. Figures 10, 11 and 12 illustrate probabilistic liquefaction hazard maps for the probability of exceedance (*PoE*) of 10% in 50 years (a return period of 475 years). These maps can be compared with Fig. 9 showing the observed liquefaction areas during the 1999 Kocaeli earthquake, which caused structural damages to buildings in Adapazari. It can be noted that the areas with high liquefaction severity in Figs. 10, 11, 12 show similarities with the areas of observed liquefaction. This is due to the fact that the PGA levels experienced in Adapazari during the 1999 Kocaeli earthquake are very similar to PGA values obtained from PSHA for *PoE* of 10% in a 50 year return period. In Fig. 12, PLHA hazard map is calculated based on unbiased $LPI$ values, where $CRR$ value is multiplied by a factor of 1.4 as proposed by Juang et al. (2005), leading to less conservative results.

### Scenario Earthquake for Adapazari (Deterministic Method)

**Fig. 13:**
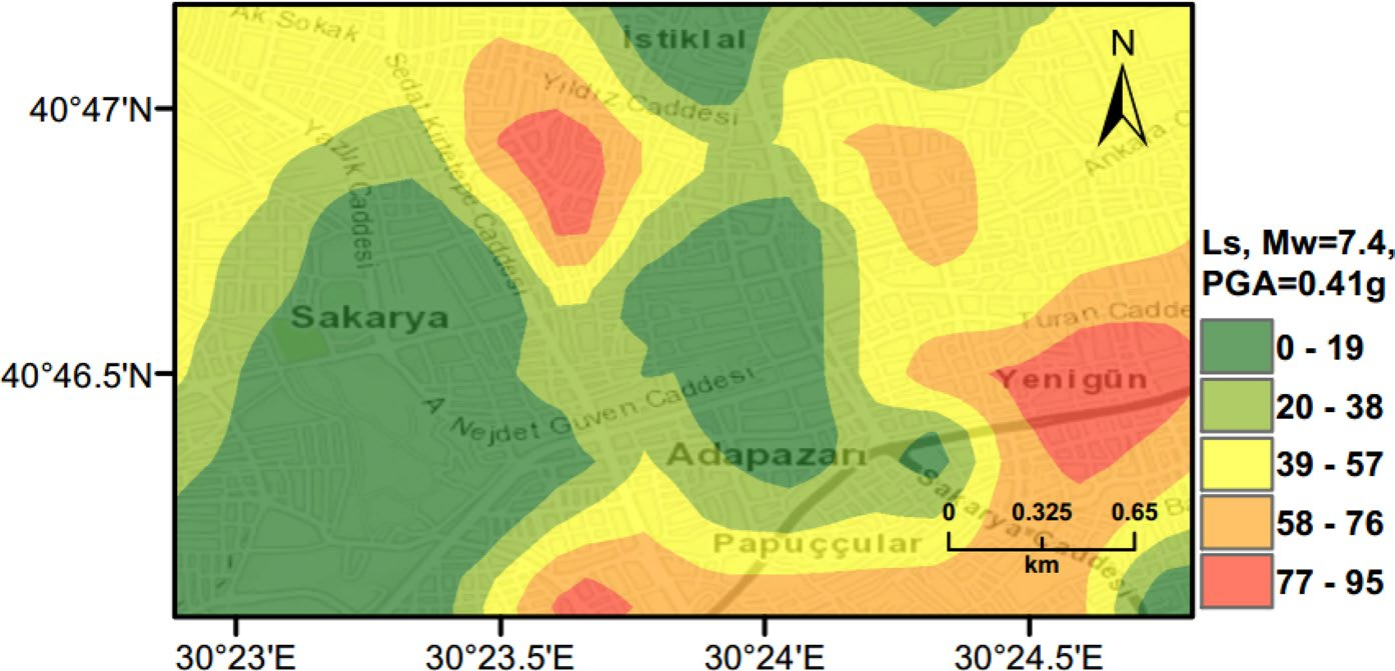
Scenario earthquake liquefaction hazard map in terms of ${L}_{S}$ for the central part of Adapazari city for *M*_*w*_ = 7.4 and PGA = 0.41 g

**Fig. 14:**
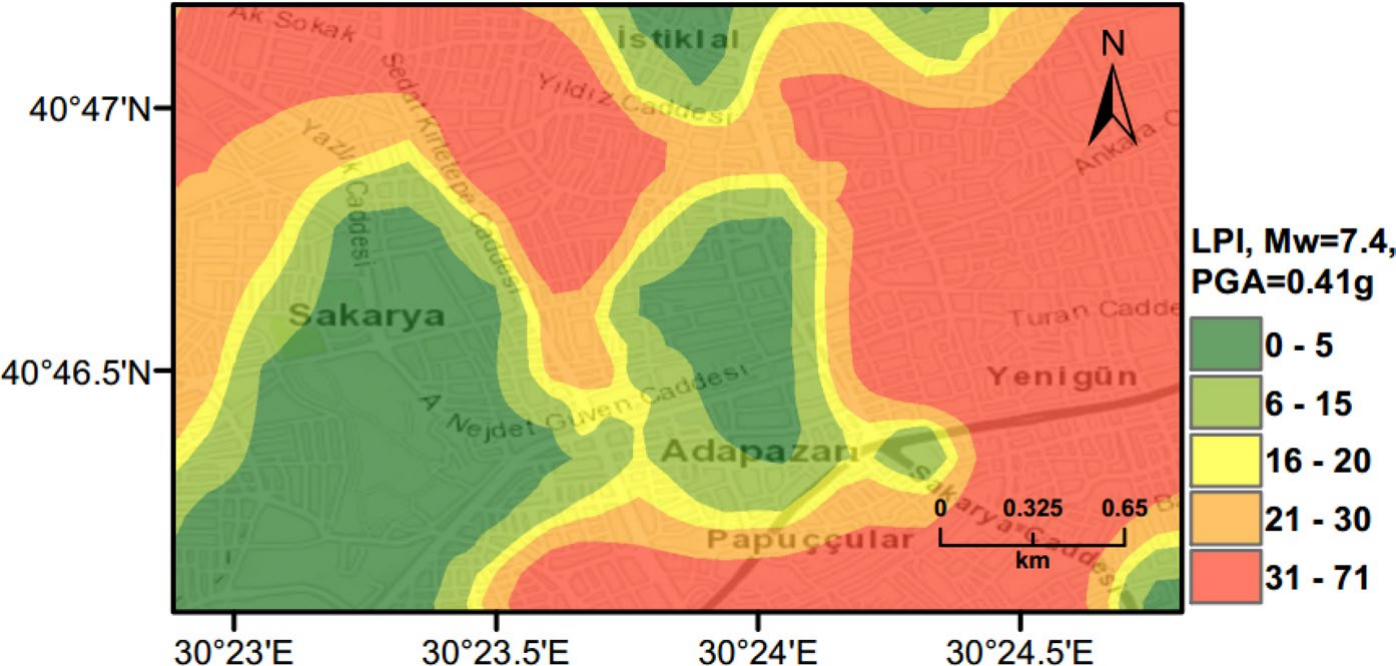
Scenario earthquake liquefaction hazard map in terms of $LPI$ for the central part of Adapazari city for *M*_*w*_ = 7.4 and PGA = 0.41 g

**Fig. 15:**
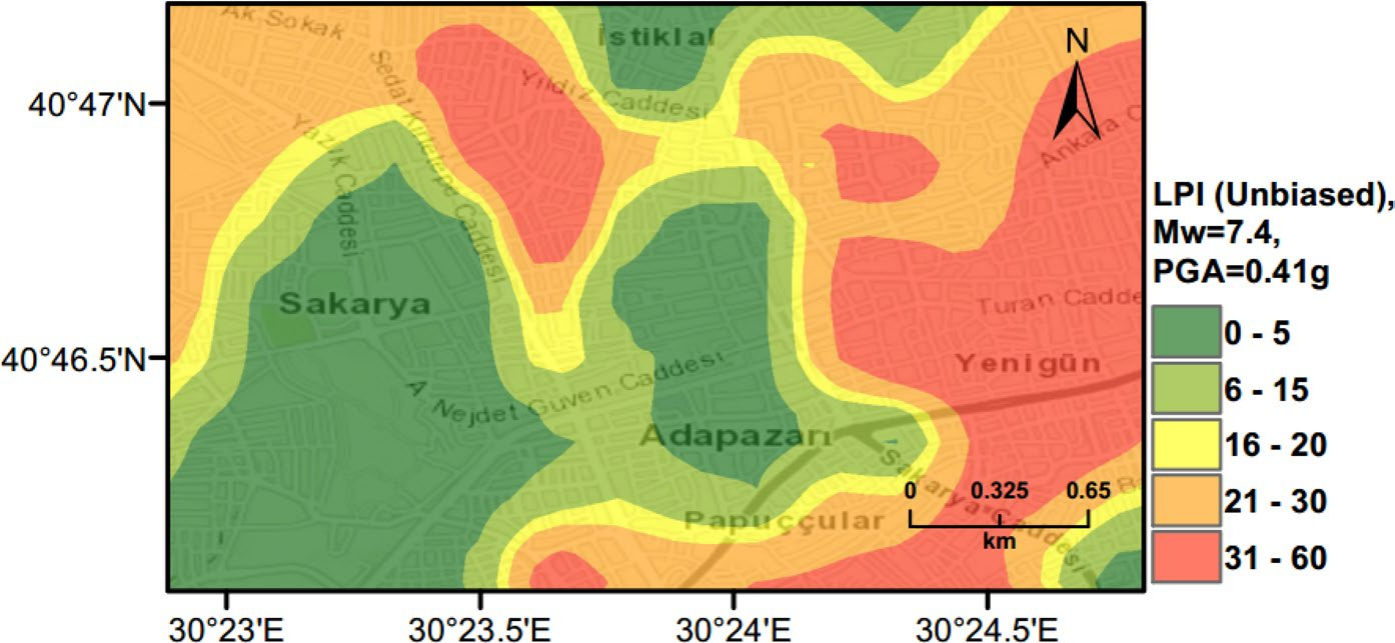
Scenario earthquake liquefaction hazard map in terms of unbiased $LPI$ for the central part of Adapazari city for *M*_*w*_ = 7.4 and PGA = 0.41 g

To show the accuracy of ${L}_{S}$ and $LPI$ hazard assessment procedures, a scenario earthquake is simulated for the city of Adapazari to compare with the liquefaction observed during the 1999 Kocaeli earthquake. An earthquake event with *M*_*w*_ = 7.4 and PGA = 0.41 g is used representing the actual values recorded during this event. Figures 13, 14, 15 show liquefaction hazard maps in terms of ${L}_{S}$ and $LPI$. For the central part of Adapazari, the predictions are well-matched to the observed liquefaction hazard from Yoshida et al. (2001). It should be noted that, Yoshida et al. (2001) investigated the liquefaction occurrence in an area slightly larger than the outer limits of the “ring” given in Fig. 9. Therefore, it is difficult to say if the predicted liquefaction is actually occurring outside of this area or not. On the other hand, if liquefaction predictions in Figs. 13, 14, 15 are compared to observations from Mollamahmutoglu et al. (2003), there are additional areas for which liquefaction occurrences match with the predictions of the proposed procedure. By comparing the deterministic hazard maps obtained from ${L}_{S}$ and $LPI$ methods with those obtained from PLHA for a return period of 475 years, it can be concluded that the probabilistic maps (Figs. 10, 11, 12) are predicting higher hazard due to the selected return period and consideration of numerous earthquake events.

**Fig. 16:**
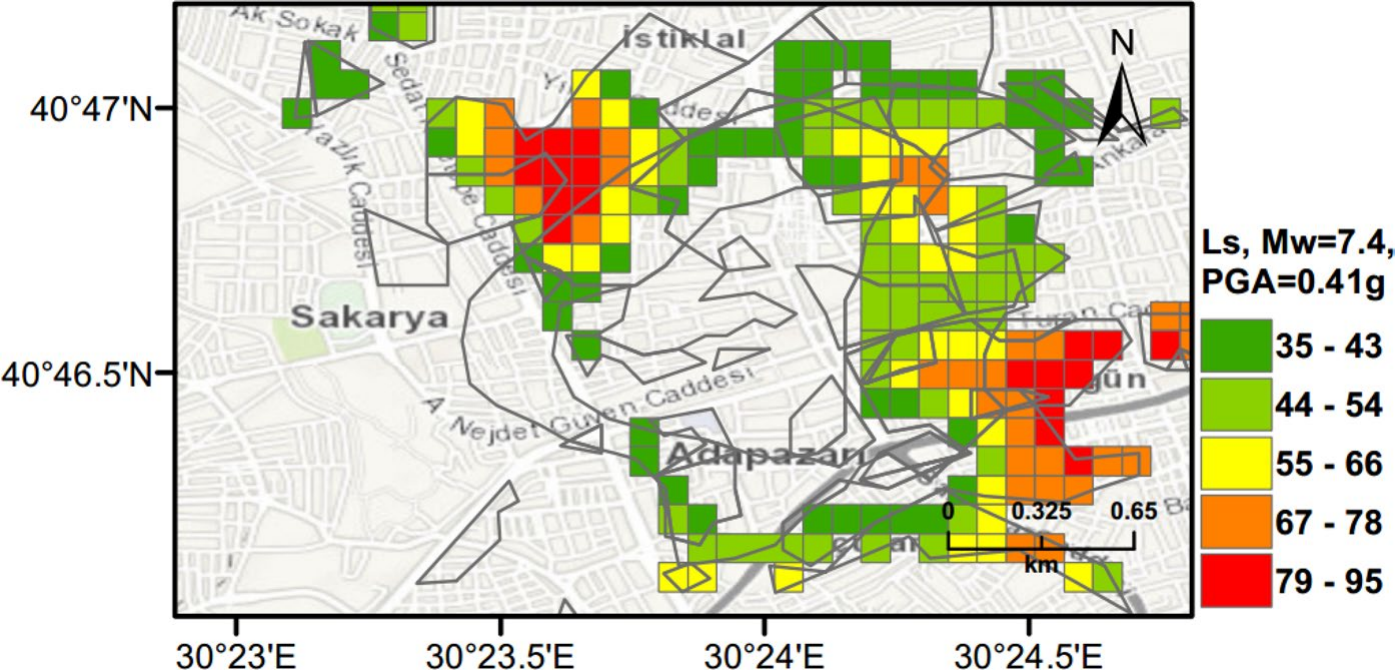
Overlapping area between liquefied area observed by Yoshida et al. (2001) and Mollamahmutoglu et al. (2003) and obtained using the ${L}_{S}$ index method with a minimum 35% threshold level (moderate severity)

**Fig. 17:**
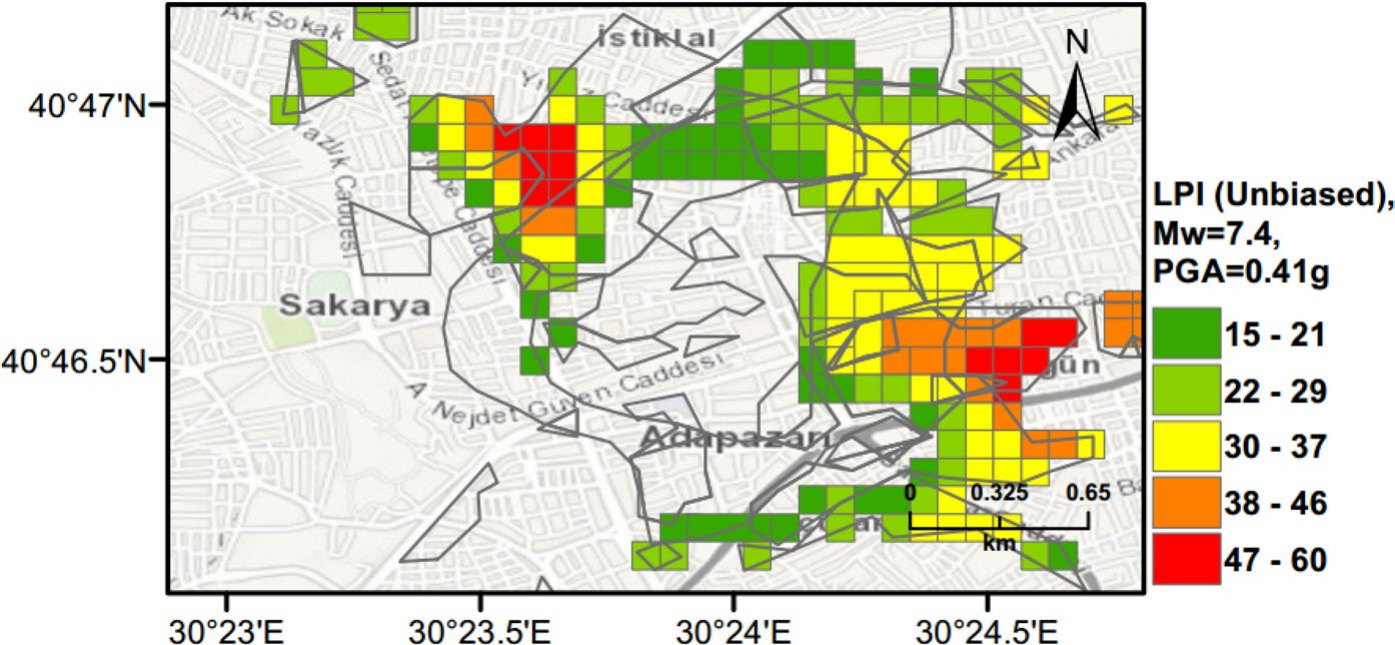
Overlapping area between liquefied area observed by Yoshida et al. (2001) and Mollamahmutoglu et al. (2003) and obtained using the unbiased $LPI$ method with a minimum threshold level of 15 (sand boils and lateral spreads)

The observed (Fig. 9) and predicted liquefaction hazard data (Figs. 13 and 15) are further evaluated to quantify the agreement between these results. Figure 16 shows the overlap between liquefied area observed by Yoshida et al. (2001) and Mollamahmutoglu et al. (2003) and obtained using the ${L}_{S}$ index method with a minimum 35% threshold level (moderate severity). For this case, the overlapped area is equal to 73% of the liquefied area. Similarly, when unbiased $LPI$ method with a minimum threshold level of 15 (sand boils and lateral spreads) is used to predict the liquefaction, the overlap area is around 72% of the liquefied area during the 1999 Kocaeli earthquake  (Fig. 17). Therefore, the proposed framework can predict liquefaction hazard with high accuracy utilising both ${L}_{S}$ and $LPI$ methods (Figs. 16 and 17), respectively.

## Small Scale Case Study: Marmara Region

**Fig. 18:**
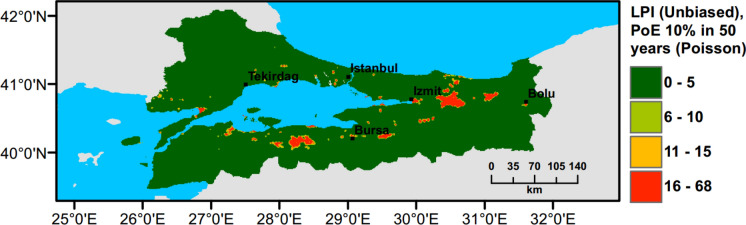
PLHA map for the Marmara region in terms of unbiased $LPI$ for a return period of 475 years based on Poisson model

**Fig. 19:**
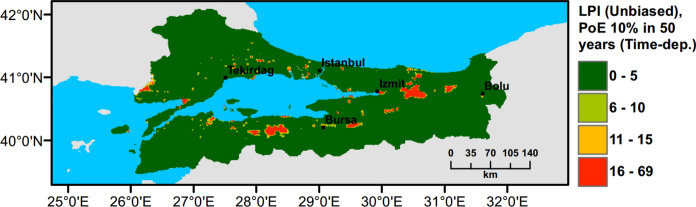
PLHA map for the Marmara region in terms of unbiased $LPI$ for a return period of 475 years based on time-dependent model

**Fig. 20:**
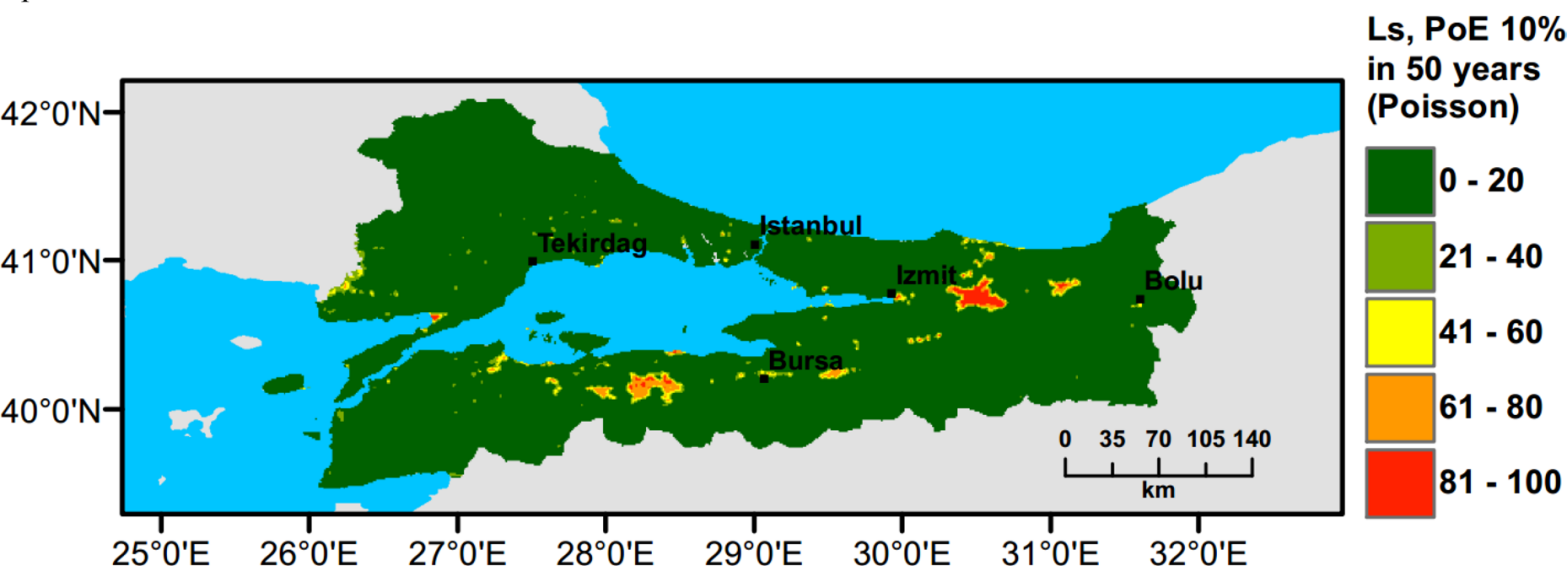
PLHA map for the Marmara region in terms of ${L}_{S}$ for a return period of 475 years based on Poisson model

**Fig. 21:**
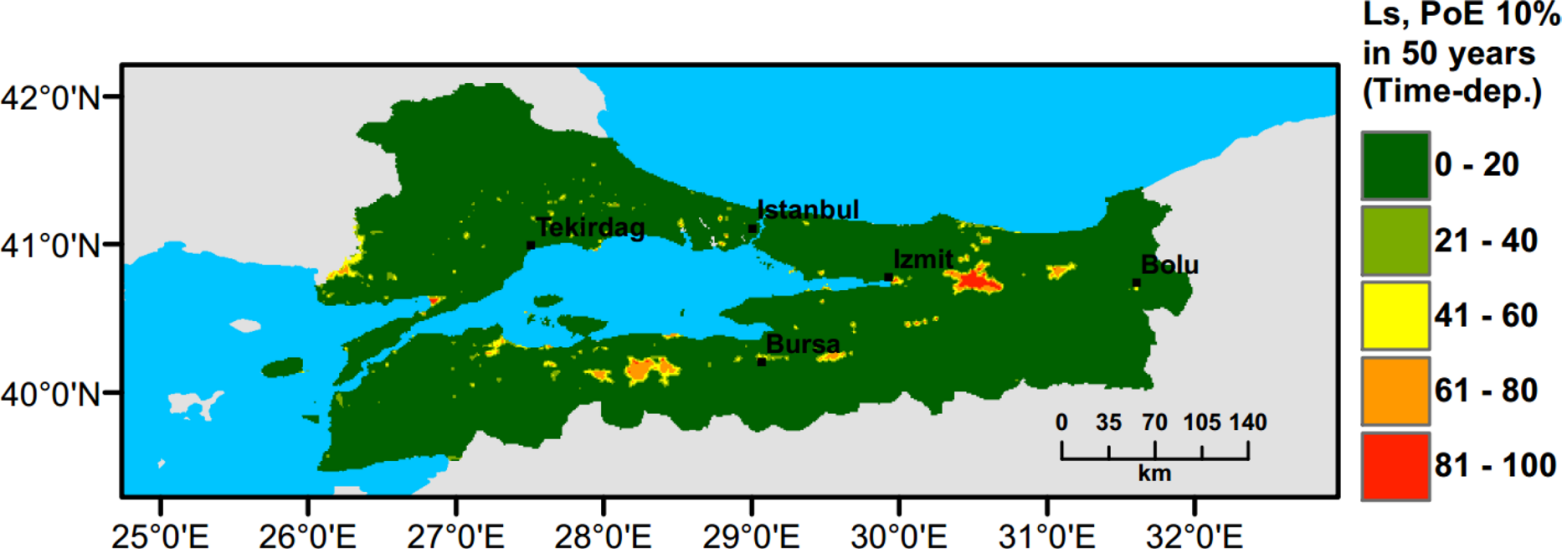
PLHA map for the Marmara region in terms of ${L}_{S}$ for a return period of 475 years based on time-dependent model

Currently, there is no liquefaction hazard map available at a regional scale for Türkiye and particularly for the Marmara region. To address this need, a set of liquefaction hazard maps for 475 years return period are prepared. Ground water level (GWT) is conservatively assumed around 1 m across the region, while slope based ${V}_{s30}$ data from USGS are adopted to perform PLHA. In Figs. 18, 19, 20, 21, $LPI$ and ${L}_{S}$ procedures are utilised considering Poisson and time-dependent (Renewal) hazard models. It can be observed that when compared to the Poisson model, in the time-dependent model liquefaction hazard more liquefaction locations are observed in the western part and slightly less in the eastern part of the Marmara region. This is due to the fact that the major faults did not rupture in the western part of the Marmara for a long period of time, while relatively recent earthquakes have occurred in the eastern part. This lowered the time-dependent seismic hazard and as a consequence reduces the liquefaction hazard in the eastern part of the region.

It should be noted that the liquefaction hazard maps developed for the Marmara region (Figs. 18, 19, 20, 21) are indicative, rather than precise, due to the lack of detailed soil data and lower resolution (~17000 data points in total) in comparison to detailed micro-zonation studies. There are a number of local liquefaction hazard studies carried out for small areas in the Marmara Region, such as Inegol area by Sonmez (2003), Bolu area by Ulamis and Kilic (2008), Izmit bay by Sonmez and Ulusay (2008) and South of lake Manyas by Kürçer et al. (2017). The results of these studies show good correlation with the liquefaction predictions shown in the PLHA maps developed by this study.

## Conclusions

In this study, a PLHA procedure based on MC simulations is proposed to develop liquefaction hazard curves and PLHA maps for seismically active regions. The developed procedure is practical and efficiently incorporates uncertainties in earthquake and soil related input parameters in a controlled way using distribution functions. A notable feature of the proposed PLHA procedure is its ability to automatically identify peak acceleration and magnitude pairs that contribute most significantly to the liquefaction hazard, eliminating the need for PSHA disaggregation. Both $LPI$ and ${L}_{S}$ liquefaction prediction methods are integrated into the framework to quantify liquefaction hazard. The procedure's efficiency is demonstrated through large and small scale case studies conducted for the city of Adapazari and the Marmara region of Türkiye, respectively. A parametric analysis investigates the impact of different stress-reduction factor ${r}_{d}$ calculation methods on liquefaction prediction parameters $LPI$ and ${L}_{S}$ and the distribution of earthquake magnitudes contributing to the liquefaction hazard. Liquefaction hazard curves and maps are developed for the city of Adapazari in terms of $LPI$ and ${L}_{S}$, while, indicative PLHA maps for the Marmara region are prepared using both time-dependent and Poisson models within the PSHA framework. The following conclusions can be drawn from the presented study:

1. The liquefaction hazard maps prepared for the city of Adapazari show good agreement with observed liquefaction following the 1999 Kocaeli earthquake. The PLHA results show that the liquefaction hazard for Adapazari can be considered as very high. The hazard maps for the city of Adapazari highlight the significant influence of ${V}_{s}$ profiles on liquefaction potential. The *r*_*d*_ method proposed by Liao and Whitman (1986) provides more conservative predictions than the method proposed by Cetin and Seed (2004), leading to a higher liquefaction hazard for the corresponding return periods.
2. A comparison between PLHA maps developed using the Poisson and time-dependent PSHA models for the Marmara region show that the time-dependent PSHA model identifies additional areas of non-negligible liquefaction hazard in the region.
3. The *r*_*d*_ procedure used in the PLHA affects the magnitude-distance distribution and the obtained $LPI$ value. Disaggregation of liquefaction hazard shows that the contribution of earthquakes with magnitude Mw < 6 to the liquefaction hazard is smaller in the *r*_*d*_ procedure proposed by Cetin and Seed (2004) than that developed by Liao and Whitman (1986). These results support Green and Bommer (2019) findings that earthquakes of magnitude smaller than M = 5 should not be considered in liquefaction hazard calculations, contrary to Musson (1998) who recommended that lower magnitudes should not be excluded from the analysis.

To conclude, the MC-based PLHA procedure proposed in this work can serve as an efficient tool for the development of liquefaction hazard curves and PLHA maps to use in performance-based design applications and for a better prediction of future hazard in loss estimation studies. The developed PLHA maps for Adapazari and the Marmara region will allow designers and decision-makers to assess the expected liquefaction hazard in these areas more accurately to reduce future earthquake related losses.

## Data Availability

Borehole logs received from Adapazari municipality are confidential. The rest of the data supporting the outcomes of this study is available within the article.

## Code Availability

The procedure described in this paper was developed using MATLAB (R2018a). Due to licensing restrictions, this code is not publicly available.
